# Supplementary material for: Sphaeroptica: A tool for pseudo-3D visualization and 3D measurements on arthropods
Source: PLoS One. 2024 Oct 23;19(10):e0311887. doi: 10.1371/journal.pone.0311887 (PMC11498740; doi:10.1371/journal.pone.0311887)
Supplement: Attachment — Submitted filename: Response to reviewer (1).docx [file pone.0311887.s001.docx]

**Response to reviewers**

Dear Editor, dear reviewers,

Thank you for giving us the opportunity to submit a revised version of the manuscript “Sphaeroptica: A Tool for pseudo-3D Visualization and 3D Measurements on Arthropods” for publication. We appreciate the time and effort that you and the reviewers dedicated to providing feedback on our manuscript and are grateful for the insightful comments and valuable improvements to our paper. We have carefully considered the reviewers’ comments and made the necessary revisions to the manuscript.

Please see below, in red, for a point-by-point response to the reviewers’ comments and concerns.

***Reviewer 1:***

*1. Although the manuscript emphasizes 3D geometric morphometrics (GM), none of the analyses directly relate to GM. Landmark coordinates are used for linear measurements, but the coordinates were not extracted and superimposed to obtain shape variables. This process could be used to compare different specimens in Procrustes shape space and determine if differences between specimens (e.g., based on Procrustes distances) are consistent when using Sphaeroptica versus microCT models.*

**Author response:** Indeed, the reviewer is correct. We did not apply 3D geometric morphometrics (GM) in this study, and therefore it should not be emphasized. We have revised the title and content to focus on the visualization tool and the linear measurements. However, we have also included a discussion on the potential applications of Sphaeroptica for GM, explaining how it could be used in future analyses.

*2. It is essential that error tests (between methods and/or observers) are also conducted using the 3D coordinates themselves. Currently, the error tests rely on linear distances between landmarks, which can be flawed. Different pairs of landmarks can have similar distances, especially in circular or elliptical structures like an insect's body. Therefore, the landmark positions may differ significantly without greatly affecting the linear measurements. This potential bias can be addressed by running error tests directly on the coordinate data. For instance, the authors could:
 - A. First, calculate the percentage of mean deviation per landmark coordinate across repetitions within the same technology/tool (Sphaeroptica, microCT) and then compare the % error between technologies
 - B. Then, also re-run the calculation% after Procrustes-superimposing the landmarks (together with those from the microCT models) to see if Procrustes distances between techniques (in%) are greater than within when they are all superimposed together.*

**Author response:** We appreciate the reviewer's insightful comment regarding the importance of error testing directly on 3D coordinates. While we acknowledge the potential benefits of this approach, our primary focus in this paper was to demonstrate the feasibility and accuracy of conducting **3D linear measurements** of arthropod structures using Sphaeroptica. We believe that by concentrating on linear measurements, we effectively showcase the tool's potential for precise morphological analysis.

In Table 11, we have evaluated the repeatability of the linear measurements over 20 measurements for each technique, using standard deviation, percentage of deviation to average and the alpha value of repeatability test of Cronbach.

Additionally, for each specimen, we have added a table representing the absolute average difference and cumulated differences for each technique over all measurements of the specimens.

We recognize the limitations of relying solely on linear distances and the potential for inaccuracies in certain cases. As such, we plan to incorporate the suggested error tests based on 3D coordinates in future studies to further refine the capabilities of Sphaeroptica.

*3. The authors should also at least discuss whether Sphaeroptica could potentially reliably digitize sliding semi-landmarks along curves or surface patches, which are increasingly used in some fields and can provide more detailed information on 3D morphological variation.*

**Author response:** Sphaeroptica cannot measure semi-landmarks at the moment. This is part of future development. We have specified this in the manuscript.

*4. The manuscript should include more specific information on the expected training time required to learn the process, whether prior knowledge of certain techniques (photogrammetry) is required, and whether observers need to be trained by someone familiar with the approach.*

**Author response:** We have added this to the manuscript.

*5. A major limitation of this new tool is its time-consuming nature compared to other techniques, related to the large amount of data generated per specimen. The authors candidly address this issue in Table 10 and Section 3.3, comparing across technologies/techniques, and suggest potential improvements. Further speeding up the processing would further enhance the practical value of this tool for analyzing larger sample sizes.* ***Considering the high number of images, would some sort of a picture-sampling algorithm help, perhaps (without affecting the reliability of measurement)?*** *- Also, I would encourage the authors to briefly discuss the applicability on larger sample sizes.*

**Author response:** We appreciate the reviewer's concerns about processing time and agree with him that it is a limitation we need to overcome in future developments. As detailed in Section 3.3, we have compared Sphaeroptica's performance to other techniques and identified areas for optimization. Nevertheless, maintaining high image quality is crucial for optimal visualisation purposes and we believe it is one of the key advantages of the proposed method. However, we are actively exploring alternative methods to expedite the process, such as speeding up the stacking process using other processing tools, as well as considering other photogrammetric softwares.

While Part 4 emphasised Sphaeroptica's potential for various specimen sizes, a comprehensive analysis of larger non-arthropod specimens presents distinct challenges that warrant dedicated investigation. To maintain the manuscript's focus, we will address this topic in a separate work.

*6. The authors mention the limitation of movement artifacts that prevent landmark-based analysis of extremities (e.g., legs). While this is addressed in the Discussion with potential plans for improvement, such as using a robotic arm, other possible solutions should be briefly discussed (e.g., perhaps after ditigizing landmarks on extremities across pictures, or by filtering out images with considerable movement).*

**Author response:** We understand the reviewer's concerns about movement artifacts affecting landmark-based analysis of extremities. As mentioned, we opted to avoid such analyses in this comparison to ensure data consistency across techniques. While movement artifacts can occur during image acquisition, with systems like ScAnt where the specimen moves, it is important to note that Sphaeroptica is a versatile tool capable of processing images from various acquisition methods, including robotic arms.

Our co-authors from HEIG-VD have developed a script to filter out images with excessive movement, which we hope to integrate into future versions of Sphaeroptica. Additionally, we will be exploring the potential of using landmark data from multiple images to mitigate the effects of movement on extremity analysis.

*7. To my understanding, each landmark can be digitized on a single 2D image. Two images = 2 landmarks to use for measuring distance. If that is the case, it would be interesting and important to have some estimate of the variability (e.g. deviation %) of the same landmark position across different consequent images (where the same structure is visible). If such error occurs between images for the same landmark (which might theoretically be possible since each 2D image itself is lacking 3D information within it), this might mean that the choice of an exact image for the landmark may decrease measuring repeatability (e.g., if Observer 1 chooses image 4500 for digitizing landmark X, but Observer 2 chooses image 4503 for the same landmark). If the error is not considerable, then it would suggest that the choice of image does not affect the result. If the authors have saved this information (which image was used) for their already performed tests, error (% deviation in landmark coordinates) should be quite fast to calculate.*

**Author response:** The reviewer raised valid concerns about landmarks accuracy, but we fear there was some misunderstanding regarding our method. We have clarified that each landmark is indeed placed on at least two images. While acknowledging the potential influence of specific image choice on landmark precision, we believe that the use of multiple images by our operators, who selected images based on ease of landmark identification, minimises this effect.

***Reviewer 2:***

*In their paper, the authors present their software “Sphaeroptica,” which facilitates 3D measurements from a sphere of 2D images, rather than from a 3D model calculated from these images. Although this might initially seem counter-intuitive, it is actually a very clever approach. This method allows for the inclusion of tiny details, such as small bristles or hairs that are visible in the photographs but often fail to be represented in a photogrammetry-based mesh.*

*Sphaeroptica is open source, with all resources and detailed manuals readily available. It works in tandem with popular photogrammetry systems like scAnt and DISC3D, enabling users to easily integrate the software into their existing workflows.*

*The authors demonstrate the effectiveness of Sphaeroptica by comparing its results with landmarking based on photogrammetry, structured light, and X-ray micro-CT. This section is detailed and documents the thorough evaluation process. The selection of test samples effectively reflects various challenges.*

*The paper is well-written, technically sound, and supported by convincing figures and tables that document the software's functionality and the evaluation results. I am convinced that Sphaeroptica is a very useful tool for anyone working on arthropod 3D geometric morphometrics. I congratulate the authors on their work and look forward to seeing this paper published.*

**Author response:** Thank you!

*Minor remarks:*

*Line 288: “Coleoptera” (capital C)*

*Line 296: “Coleoptera” (capital C); “Ovalisia” (italics); “sp.” (no italics)*

*Line 318: “and” (no italics)*

**Author response:** Thank you to the reviewer for correcting these minor errors. All have been corrected within the paper.

*Line 685: Consider using a different abbreviation than “CT,” as this usually stands for “computed tomography” and may cause confusion.*

**Author response:** We agree with the reviewer that this abbreviation can be confusing. As a consequence, we switched from “CT” to “PT”, which stands for “processing time”. We hope this adds clarity to the text.
